# Characterization of Nutritional and Bioactive Compound in Three Genotypes of Mashua (*Tropaeolum tuberosum* Ruiz and Pavón) from Different Agroecological Areas in Puno

**DOI:** 10.1155/2022/7550987

**Published:** 2022-03-23

**Authors:** Alejandro Coloma, Emilio Flores-Mamani, German Quille-Calizaya, Arturo Zaira-Churata, Jorge Apaza-Ticona, Wilber César Calsina-Ponce, Percy Huata-Panca, Juan Inquilla-Mamani, Félix Huanca-Rojas

**Affiliations:** Universidad Nacional del Altiplano - Puno, Peru

## Abstract

Bioactive compounds and nutritional characterization in three genotypes of mashua tubers (*Tropaeolum tuberosum* Ruiz and Pavón) from different agroecological areas of the Peruvian highlands (3747 at 3888 meters above sea level) were studied. The studied genotypes from different agroecological areas significantly differed for vitamins, amino acids, and bioactive compounds. However, the nutritional characteristics of yellow, purple, and yellow-purple mashua remained unaffected. Its tubers were shown to be important sources of protein and fiber. The nutritional analysis revealed high phosphorus and potassium values, as well as considerable amounts of vitamin C. The amounts of total free amino acids in the genotypes ranged from 2.73 ± 0.450 mg/g dry matter (DM) to 6.825 ± 0.450 mg/g DM. Important total anthocyanins, total flavonoids, total phenolics, tannin content, and antioxidant activity values were obtained from purple genotype. Unexploited colored mashua tubers are proposed as a valuable natural source of phenolics and anthocyanins with high antioxidant activity.

## 1. Introduction

Mashua (*Tropaeolum tuberosum* R. & P.) is a perennial herbaceous plant, belonging to the Tropaeolaceae family [1]. This crop is known as cubio in Colombia [2], mashua in Perú [3, 4] and Ecuador [5], and isaño or añu in Bolivia [6]. The tuber originated in Peru and Bolivia and has grown for thousands of years, but its cultivation has extended to other countries out of the plateau as well as Ecuador, Venezuela, Colombia, Argentina, and in many regions of New Zealand, Canada, the United States, and England [7]. Currently, it is grown in small cultivation plots in the high Andean areas; it has a high genetic diversity. Tubers can present many colors such as yellow, purple, and white. They are cooked after being left out in the sun to improve their flavor [8]. It is grown alongside other tubers (potato, oca, and olluco), so it is difficult to know its level of production [5].

They are consumed mainly by the indigenous population as a part of their daily diet and on special occasions [5], where it is popularly believed to possess nutritional and medicinal properties [1]. They have recently gained the world attention due to the wide range of nutrients and phytochemicals they possess [9]. Therefore, this tuber could be considered as novel and inexpensive sources of bioactive compounds for its potential use in functional foods and nutraceuticals [10]. This species is rich mainly in polyphenols, flavonoids, anthocyanins, antioxidants, fatty acids, glucosinolate, and alkamides [7, 11, 12]. Mashua has been tested for various biological activities, and the extract (tuber juice) demonstrated a promising potential as an antibacterial, antioxidant, anti-inflammatory, and inhibitors of benign prostatic hyperplasia [1]. It is used to treat venereal, lung, and skin diseases, as well as to heal wounds and as an analgesic for kidney and bladder pain [11–13].

There is a growing interest in natural sources of nutrients and health-promoting compounds. The nutritional value and bioactive components of mashua have been collected by Pérez and Apaza [11] and Guevara-Freire et al. [7]. Macronutrient contents vary greatly depending on the origin, variety/ecotype, environmental conditions, geographical area, and composition of the soil for cultivation they possess [14]. The agroecological area of the Peruvian highlands is large and complex. It contains a wide variability of agricultural production determined by specific physical factors such as the relief of the soil surface, the type of soil, and its hydric characteristics [15]. This variability in terms of color and shape seems to be correlated to the content of bioactive compounds they possess [14]. Our hypothesis is that the agroecological areas lead to changes in physicochemical characteristics and bioactive compounds in three genotypes of mashua. Thus, it is proposed to evaluate the effect of agroecological areas of production on the physicochemical characterization and content of bioactive compounds in three genotypes of mashua.

## 2. Materials and Methods

### 2.1. Plant Material

**Figure 1:**
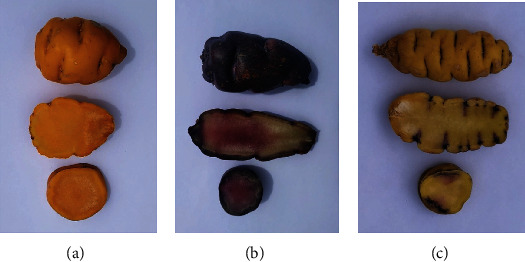
Genotypes of mashua tubers: (a) yellow, (b) purple, and (c) yellow-purple.

Three genotypes of mashua tubers (Figure 1), yellow, purple, and yellow-purple (yellow with purple eyes) known as isaño, were collected from the local fairs in four agroecological areas of the region of Puno (Perú), El Collao (Maquercota hamLet, 3747 m.a.s.l., latitude 16° 05′31.0^″^ S and longitude 69° 30′ 27.2^″^ W), Chucuito (Molino hamLet, 3888 m from the sea level (m.a.s.l.), latitude 16° 13′ 38.4^″^ S and longitude 69° 23′ 17.7^″^ W), Yunguyo (Sanquira hamLet, 3847 m.a.s.l, latitude 16° 19′ 21.4^″^ S and longitude 69° 03′ 03.0^″^ W), and Puno (Paucarcolla district, 3810 m.a.s.l, latitude 15° 46′ 06.3^″^ S and longitude 70° 04′ 05.0^″^ W), in August 2019. Botanical identification was carried out by the Botany Laboratory of the National University of Altiplano Puno (Peru). Harvested tubers were immediately packed in paper bags and sent to the Chromatography and Spectrophotometry Laboratory of the Faculty of Sciences of the National University of San Antonio Abad (Cusco, Peru) where they were lyophilized and stored at −20°C. Three samples made of 10 tubers of each genotype were randomly collected for analysis. The lyophilized samples were rehydrated prior to the analyses to improve the recoveries of the extraction procedures [16]. All analyses were conducted in triplicate.

### 2.2. Chemicals and Reagents

HPLC-grade acetonitrile (CH_3_CN), Folin-Ciocalteu phenol reagent, aluminum trichloride, hydrochloric acid, and sodium carbonate were purchased from Merck (Darmstadt, Germany). Methanol (MeOH), ethanol, 2-propanol, potassium acetate, orthophosphoric acid, sodium dihydrogen phosphate, potassium hydroxide, and hexane were obtained from J.T. Baker Chemical Co. (Phillipsburg, NJ, USA). Tannic acid, quercetin, gallic acid, and 2,2-diphenyl-1-picrylhydrazyl (DPPH) were provided from Sigma-Aldrich Chemical Co. (St. Louis, MO, USA). Trolox was supplied by Santa Cruz Biotechnology. L-Ascorbic acid and amino acid standard 17 types were procured from Sigma-Aldrich Chemical Co. (St. Louis, MO, USA).

### 2.3. Proximal Composition

AOAC methods were used to examine mashua tuber: moisture content was determined by the drying method using hot-air oven circulation (method #925.09). Ash content of a known weight sample was determined through incineration (550°C) using a muffle furnace (method #923.03). Crude protein was determined by micro-Kjeldahl (method #979.09) and calculated by multiplying the corresponding total nitrogen content by a factor of 6.25.The crude fat content of the sample was determined by a Soxhlet extractor (method #930.09). Crude fiber content was determined by the following method #962.09. Carbohydrate content is determined by subtracting the total percentage of other components from 100 [16]. The results were expressed as g/100 g of dry matter (DM).

### 2.4. Mineral Analysis

The samples were dried at 105°C for 24 h. Dried samples were homogenized using an agate homogenizer and stored in precleaned polyethylene bottles until analysis. 0.25 g each of the powdered plant samples was digested in 6.5 mL of acid solution (HNO_3_, H_2_SO_4_, and HClO_4_ in a ratio of 5 : 1 : 0.5). The corresponding solution was heated until white fumes had appeared. The clear solution was diluted up to 50 mL with distilled water and filtered with Wattman filter paper no. 1. The standard working solutions of elements of interest were prepared to make the standard calibration curve. Absorption for a sample solution uses the calibration curves to determine the concentration of particular element in that sample. Calcium was determined by EDTA titration method [17], iron was determined by photometry with 1,10-phenanthroline [18], phosphorus was determined by spectrophotometry with phosphomolybdate complex [19], potassium was determined by spectrophotometry with cobaltinitrite of sodium [20], and a Varian AA240FS atomic absorption spectrometer (AAS) was used for the determination of zinc [21].

### 2.5. Vitamin Analysis

#### 2.5.1. *β*-Carotene Content Determination

*β-carotene* was extracted according to the procedure of Rivera and Canela [22]. One gram of mashua genotype was extracted with 3 mL of 2-propanol (solvent/material ratio: 3/1) in an orbital shaker (Heidolph, Schwabach, Germany) at 200 rpm under darkness for 30 min at room temperature (20°C). The mixtures were centrifuged at 2000 × *g* for 10 min at 4°C and the supernatants were collected. The mashua residues were reextracted three more times under the same conditions. The supernatants of each extraction were filtered through a 0.45 *μ*m Sartolon polyamide filter and degassed by sonication prior to use. A 400 mg/L of *β*-carotene was prepared by dissolving a 0.0100 g of *β*-carotene in hexane and made up to volume of 25 mL.

The extract of mashua and standard solutions were then analyzed by an Agilent Technologies 1200 Series HPLC (Agilent Technologies, Palo Alto, CA), equipped with an Agilent 1200 HPLC variable wavelength detector. The chromatographic separation was performed on a Zorbax Eclipse SB-C18 analytical (75 × 4.6 mm i.d., particle size 3.5 *μ*m) column with guard column Zorbax Eclipse XDB-C18-Pack (12.5 × 4.6 mm i.d., particle size 5 *μ*m) both purchased from Agilent Technologies. Detection was made at a wavelength of 450 nm, and the column oven temperature was set at 25°C. The injection sling was 1.0 *μ*L. The mobile phase consisted of methanol/2-propanol (50 : 50, *v*/*v*). The mobile phase was prepared daily, filtered and sonicated before use, and delivered at a flow rate of 1.0 mL/min. Quantification of *β*-carotene was based on peak areas and calculated as equivalents of standard compounds. Different concentrations of unknown samples of *β*-carotene were determined using the obtained regression equations. Total contents were expressed as milligram per g dry matter (DM).

#### 2.5.2. Vitamin C Content Determination

Vitamin C was extracted according to the procedure of Campos et al. [23] with some modifications made to it. Briefly, 0.5 g of mashua was mixed with 1.5 mL of H_3_PO_4_ 4.5%. The mixture was homogenized with a mortar, 1.5 mL of water was added and then was centrifuged at 4000 × *g* for 10 min, and the supernatants were collected. The supernatants of each extraction were filtered through a 0.45 *μ*m Sartolon polyamide filter and degassed by sonication prior to use at 4°C. Aliquots from stock standard solutions (0.1 mg/mL) of AA were transferred into a series of 10 mL volumetric flasks. The contents of each flask were completed with the mobile phase to volume to get a concentration range of 0.5-10.0 *μ*g/mL. This solution was kept in darken bottle at 4°C. The extract of mashua and standard solutions were then analyzed by an Agilent Technologies 1200 Series HPLC (Agilent Technologies, Palo Alto, CA), equipped with an Agilent 1200 HPLC variable wavelength detector.

The chromatographic separation was performed on a Zorbax Eclipse XDB-C18 analytical (250 × 4.6 mm i.d., particle size 5 *μ*m) column with guard column Zorbax Eclipse XDB-C18-Pack (12.5 × 4.6 mm, i.d., particle size 5 *μ*m) both purchased from Agilent Technologies. Detection was made at a wavelength of 270 nm, and the column oven temperature was set at 25°C. The injection sling was 1.0 *μ*L. The mobile phase consisted of ammonium acetate buffer 100 mM/acetonitrile (90 : 10, *v*/*v*), pH adjusted to 6.3 by orthophosphoric acid. The mobile phase was prepared daily, filtered and sonicated before use, and delivered at a flow rate of 1.0 mL/min. Quantification of the vitamin C was based on peak areas and calculated as equivalents of standard compounds. Different concentrations of unknown samples of AA were determined using the obtained regression equations. Total contents were expressed as milligram per g dry matter (DM).

#### 2.5.3. Amino Acid Determination

A method described by Park et al. (2014) was used to determine amino acid in mashua tubers. One gram was extracted with 5 mL HCl 6 N. The solution was vigorously vortexed for 5 min; air was expelled with N_2_ and hydrolyzed at 100°C for 24 h and then centrifuged at 4000 × *g* for 15 min. 1 mL of supernatant was diluted with 1.66 mL of ultrapure water and filtered through a 0.45 *μ*m PTFE syringe filter (Advantec DISMIC-13HP, Toyo Roshi Kaisha, Ltd., Tokyo, Japan). The filtrate was then analyzed by an Agilent Technologies 1200 Series HPLC (Agilent Technologies, Palo Alto, CA), equipped with an Agilent 1200 HPLC variable wavelength detector.

The chromatographic separation was performed on a Zorbax Eclipse AAA rapid resolution (75 × 4.6 mm i.d., particle size 3.5 *μ*m) column purchased from Agilent Technologies. Detection was made at a wavelength of 262 and 338 nm, and the column oven temperature was set at 35°C. The injection sling was 10 *μ*L. The solvent system was delivered at a rate of 2.0 mL/min and consisted of a mixture of (A) sodium dihydrogen phosphate buffer (40 mM) at a pH of 7.8 and (B) acetonitrile/methanol/water (45 : 45 : 10, *v*/*v*/*v*). Fifty pmol/*μ*L content of amino acids was used as standard. The gradient program used was as follows: 0–1.9 min, 0% B; 2–21 min, 57% B; 21.1–25 min, 100% B; and 25.1–30 min, 0% B. Quantification of the different amino acids was based on peak areas and calculated as equivalents of standard compounds. All contents were expressed as milligram per g DM.

### 2.6. Bioactive Compound Analysis

#### 2.6.1. Extraction of Phenolic Compounds and Antioxidants

About 2 g (accurately to 0.0001 g) of raw mashua was extracted twice with 5 mL of 70% ethanol and then homogenized in a morter. The mixtures were centrifuged at 4000 × *g* for 10 min; the supernatants were collected and stored in an amber bottle at 4°C for the analysis.

#### 2.6.2. Total Anthocyanins (TA)

TA in mashua extract was determined by using the pH differential method [24]. Absorbance was measured at 520 and 700 nm in pH 1.0 and 4.5 buffers. A molar extinction coefficient of 26,9001 cm^−1^ mol^−1^ and a molecular matter of 449.2 were used for anthocyanin calculation [25]. The results were expressed as mg of cyanidin 3-glucoside equivalents (CGE)/g DM.

#### 2.6.3. Total Flavonoids (TF)

The aluminum chloride colorimetric method was modified from the procedure reported by [26]. Quercetin was used to make the calibration curve. Ten milligrams of quercetin were dissolved in 80% ethanol and then diluted to 2.5, 5.0, and 10.0 *μ*g/mL. The diluted standard solutions (500 *μ*L) were separately mixed with 1.5 mL of 95% ethanol, 100 *μ*L of 2% aluminum chloride, 100 *μ*L of 1 M potassium acetate, and 4.5 mL of distilled water. After incubation at room temperature for 30 min, the absorbance of the reaction mixture was measured at 415 nm with a spectrophotometer (Genesys 20, Thermo Scientific, Mississauga, Ontario, Canada). The amount of 10% aluminum chloride was substituted by the same amount of distilled water in blank. Similarly, 500 *μ*L of ethanol extracts was reacted with aluminum chloride for determination of flavonoid content as described above. The results were expressed as mg of quercetin equivalents (QE)/g DM.

#### 2.6.4. Total Phenolics (TP)

The Folin-Ciocalteu method [27] was used to determine total phenolic content. 50 *μ*L obtained extract was then mixed with 100 *μ*L of 0.2 N Folin-Ciocalteu reagent (Sigma-Aldrich Chemie, Steinheim, Germany) for 5 min. Following the addition of 200 *μ*L of 20% sodium carbonate (Na_2_CO_3_) (Labosi, Paris, France), solution tubes were mixed and the final volume was completed to 1700 *μ*L with distilled water. After incubation at room temperature for 30 min, the absorbance of the reaction mixture was measured at 765 nm against an ethanol 70% blank with a spectrophotometer (Genesys 20, Thermo Scientific, Mississauga, Ontario, Canada). Gallic acid (Sigma-Aldrich Chemie, Steinheim, Germany) (0–10 *μ*g/mL in ethanol 70%) was used as standard to produce the calibration curve. The mean of three readings was used, and the total phenolic content was expressed in mg of gallic acid equivalents (GAE)/g DM.

#### 2.6.5. Antioxidant Activity

The extracts obtained above were used to assess the antioxidant activity by the DPPH (2,2-diphenyl-1-picrylhydrazyl) radical scavenging method [27]. A 50 *μ*L obtained extract was mixed with 1.5 mL methanolic solution of DPPH (100 mM). The mixture was shaken vigorously and allowed to stand at room temperature for 30 min. The absorbance was measured at 517 nm using a spectrophotometer (Genesys 20, Thermo Scientific, Mississauga, Ontario, Canada). The antioxidant activity was calculated based on a standard curve obtained with an ethanolic solution of Trolox (6-hydroxy-2,5,7,8-tetramethylchroman-2-carboxylic acid) at different concentrations. The results were expressed as *μ*mol Trolox equivalents (TE)/g DM.

#### 2.6.6. Tannin Content Determination

Tannin was extracted according to the procedure of Durgawale et al. [28]. Approximately 1 g of mashua tuber was extracted with 3 mL of mixed solvents (methanol/acetone/water, 45/45/10, *v*/*v*/*v*). The mixtures were centrifuged at 4000 × *g* for 10 min. The supernatant was filtered through a 0.45 *μ*m filter and analyzed by HPLC. Tannic acid (10 mg) was dissolved in 10 mL of mobile phase to prepare stock solution with concentration of 1000 *μ*g/mL. A series of dilutions with concentration of 20, 30, 40, and 50 *μ*g/mL were prepared by taking aliquots of 0.2, 0.3, 0.4, and 0.5 mL of stock solution (1000 *μ*g/mL) and diluted up to 10 mL with mobile phase. Each dilution was analyzed by an Agilent Technologies 1200 Series HPLC (Agilent Technologies, Palo Alto, CA), equipped with an Agilent 1200 HPLC variable wavelength detector.

The chromatographic separation was performed on a Zorbax Eclipse XDB-C18 analytical (250 × 4.6 mm i.d., particle size 5 *μ*m) column with guard column Zorbax Eclipse XDB-C18-Pack (12.5 × 4.6 mm, i.d., 5 *μ*m) both purchased from Agilent Technologies. Detection was made at a wavelength of 280 nm, and the column oven temperature was set at 40°C. The injection sling was 1.0 *μ*L. The solvent system was delivered at a rate of 1.0 mL/min and consisted of a mixture of (A) water/acetic acid (98 : 2, *v*/*v*) and (B) methanol. The gradient program used was as follows: 0–4.9 min, 5% B and 5–10 min, 50% B. Quantification was carried out using an absolute calibration curve method with standard solutions of tannic acid. The total contents were expressed as milligram per g DM.

#### 2.6.7. Statistical Analysis

Data was statistically treated by analysis of variance (ANOVA); the means were compared by the Tukey test at a significance level of 0.05 using Infostat Statistical Software. All the measurements were carried out in triplicate.

## 3. Results and Discussion

### 3.1. Composition of Chemicals

A proximal composition for three genotypes of mashua cultivated in four agroecological areas is provided in Table 1. The protein content was ranged from 6.96 ± 0.09 to 11.72 ± 0.05 g/100 g DM. The protein content is higher in purple genotype (7.41 ± 0.01 to 11.72 ± 0.05 g/100 g DM), compared to yellow genotype (6.96 ± 0.09 to 9.98 ± 0.02 g/100 g DM) and yellow-purple (7.15 ± 0.04 to 7.95 ± 0.04 g/100 g DM). The protein determined in the study was similar to the results found for mashua (9.21 ± 0.18 g/100 g DM) and olluco (8.06 ± 0.07 g/100 g DM), but higher than that of oca (6.84 ± 0.07 g/100 g DM) [29]. Mashua is a food that is highly nutritional, characterized by containing a high level of protein of elevated biological value with an ideal balance of essential amino acid [7, 11]. The nutritional quality of a protein source varies substantially depending upon their bioavailability, digestibility, amino acid profile, purity, antinutritional factors, and processing effects.

The main constituent of the dry matter in mashua was represented by the carbohydrate content (73.79 ± 0.07 to 77.50 ± 0.17 g/100 g DM). The fraction of carbohydrates consists mainly of starch, in the form granules, whose components are amylase 27% and amylopectin 73% [7]. Starch can be used for the microencapsulation of bioactive compounds as it has good viscoelastic properties [30].

The fiber content was ranged 5.23 ± 0.03 to 6.36 ± 0.03 g/100 g DM. This range is similar (6.9 g/100 g DM) to those reported by Choquechambi et al. [31]. From a nutritional point of view, this value of fiber might be helpful in terms of maintaining positive effect on intestine and colon physiology, besides other homeostatic and therapeutic functions in human nutrition [32]. Dietary fiber is known to have beneficial health effects, relieving functional constipation, a common gastrointestinal problem in children [33].

The analysis of the mineral content is presented in Table 1. The high quantity of potassium and phosphorus was obtained from mashua tubers, while the low zinc and iron were detected. The potassium content in each genotype was affected by the place of origin; in yellow mashua, it ranged between 1723.42 ± 54.55 and 2021.15 ± 5.45 mg/100 g DM, in purple from 1729.18 ± 32.77 to 1875.61 ± 15.38 mg/100 g DM, and in yellow-purple from 1743.54 ± 9.13 to 1789.77 ± 11.64 mg/100 g DM. The potassium content was the highest to the values reported (1484 mg/100 g of DM) previously, and this tuber is ideal for hypertensive people [11].

**Figure 2:**
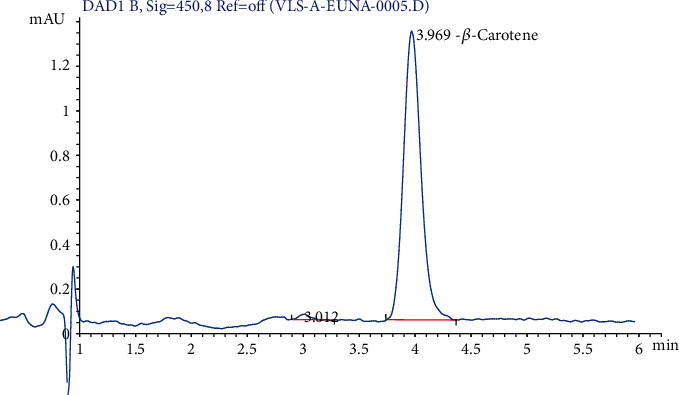
Sample HPLC chromatogram of *β*-carotene at 450 nm.

*β*-Carotene of three genotypes of mashua cultivated in four agroecological areas was analyzed (Table 1). The agroecological areas affected the *β*-carotene content of mashua, with the highest content found in the agroecological areas of Chucuito followed by Puno, Collao, and finally Yunguyo (Figure 2). The yellow genotype presented the highest amount of *β*-carotene (18.10 ± 0.13 to 715.95 ± 84.03 *μ*g/g DM), followed by yellow-purple (6.91 ± 0.03 to 336.21 ± 3.21 *μ*g/g DM). The purple one presented very little amount of *β*-carotene (5.65 ± 0.80 *μ*g/g DM). These results were similar to those reported (125 *μ*g/g DM) by Pérez and Apaza [11]. This variation could be mainly due to differences in geographic locations [34]. *α*-Carotene, *β*-carotene, and *β*-cryptoxanthin are provitamin A carotenoids, meaning they can be converted by the body to retinol. Natural vitamin A exists only in animal tissues; in vegetables, it is found as provitamin A in the form of carotenes, which is transformed into vitamin A in the human body [35]. Vitamin A is a fat-soluble vitamin; these organic molecules cannot be produced in our body and therefore should be taken from foods with daily diet [36]; they are also needed for growth, vision, reproduction, production of enzymes, and differentiation of epithelial cells [37].

In Table 1, it is shown that the agroecological areas affected the content of vitamin C in the three genotypes of mashua. The purple presented the highest amount of vitamin C (1.21 ± 0.002 to 4.46 ± 0.01 mg/g DM), followed by yellow-purple (0.90 ± 0.003 to 3.36 ± 0.002 mg/g DM), and finally, the yellow presented very little amount of vitamin C (0.53 ± 0.002 to 1.54 ± 0.0001 mg/g DM). The amount of ascorbic acid was similar to that reported by Campos et al. [9] and Pérez and Apaza [11] (4.80 mg/g DM and 2.0 mg/g DM, respectively). The mashua tuber presents important contents of vitamin C in comparison with other tuber crops [38]. Vitamin C is important in the human diet as a nutritive substance and is also a powerful antioxidant effective against oxidative stress, and it is vital for the growth and maintenance of healthy bones, teeth, gums, ligaments, and blood vessels [39, 40]. Vitamin C is very unstable, and it is drastically lost during storage [39], freezing [41], and cooking [42].

### 3.2. Amino Acid Compound

Seventeen amino acids in total, essential and nonessential, were quantified in the purple, yellow, and yellow-purple mashua genotypes (Table 2). The agroecological areas affected the total amino acid content of three genotypes, with the highest content found in the agroecological area of Yunguyo following Puno and finally Collao. The amounts of total free amino acids in purple ranged from 3.592 ± 0.320 mg/g DM to 4.659 ± 0.355 mg/g DM, whereas in yellow genotype, the highest levels of amino acids were detected in Yunguyo (6.825 ± 0.45 mg/g DM) and lowest in Collao (2.763 ± 0.42 mg/g DM); likewise in yellow-purple, the highest levels of amino acids were detected in Ilave (4.032 ± 0.232 mg/g DM) and lowest in Yunguyo (3.478 ± 0.132 mg/g DM). This result was similar to that reported by [31].

Nine essential amino acids, including histidine, isoleucine, leucine, lysine, methionine, phenylalanine, threonine, tryptophan, and valine, cannot be synthesized in the human organism and must be provided in the diet [43] and must have through the diet contributed 5 to 13% of the total free amino acids [44], amongst that yellow with purple mashua genotype showed highest percentages of amino acid accumulation. The content of individual amino acids varied considerably in purple, yellow, and yellow with purple genotypes, according to the location. Vegetables are good sources of all essential amino acids required for humans, but same vegetables may contain the trace amount to essential amino acids [44].

Among the different genotypes such as violet, yellow, and yellow with violet eyes, they contain comparatively higher amounts of isoleucine and lower amounts of phenylalanine aromatic amino acids. Valine is found in similar reports [11], while in other Andean tubers, phenylalanine+tyrosine was found in olluco and leucine in oca [45]. High levels of isoleucine were found in purple mashua from the agroecological area of Chucuito (15.42 ± 0.31 mg/100 g FW), followed by yellow from Yunguyo (13.40 ± 0.28 mg/100 g FW) and yellow-purple from Collao (13.34 ± 0.59 mg/100 g). Leucine, isoleucine, and valine are branched chain amino acids (BCAA), are critical to human life, and are particularly involved in stress, energy, and muscle metabolism [46].

The catabolism of these three amino acids is controlled by a common flux-generating step, their catabolic disposal occurs largely in the skeletal muscle, their circulating concentrations can influence the brain uptake of precursor amino acids for neurotransmitter synthesis, and they can regulate protein synthesis in a variety of tissues [47]. Only recently, supplemental amounts of branched chain amino acids (BCAA), including leucine (Leu), isoleucine (Ile), and valine (Val), are shown to accelerate a recovery from muscle damages, soreness, and fatigues after exercise [48]. Furthermore, free amino acids contribute to the taste of vegetables, like sweetness because of the presence of glycine and alanine and bitterness because of the valine and leucine, and aspartic acid and glutamate have sour tastes [44]. It is evident that a large amount of variation exists in amino acid quality. It is also evident from our job that the limiting amino acids in these tubers consumed as a complete food are valine and tryptophan [45].

Amino acids are building blocks of proteins and polypeptides [49]. In plants, the amino acids are also involved in a plethora of cellular reactions, and therefore, they influence a number of physiological processes such as plant growth and development, intracellular pH control, generation of metabolic energy or redox power, and resistance to both abiotic and biotic stress [50]. In humans, the amino acids are key regulators of gene expression and the protein phosphorylation cascade and act as precursors for synthesis of hormones and low-molecular matter nitrogenous substances with enormous biological importance such as nitric oxide, polyamines, glutathione, and taurine [51]. Among all the amino acids, some may be synthesized in the body, while others cannot be synthesized in the body at a rate necessary for normal growth and hence must be supplied in the diet [52]. Protein quality is determined by digestibility and by its content of essential amino acids [53]. The amino acid needs of the body vary with age, health status, energy balance, and physiological conditions [54]. Free amino acids (FFA) from plants can have a dual role in the diet. They are basic components of proteins and polypeptides, but they can also react with other components of the diet to give a sweet flavor to humans, such as when FFA react with free sugars during heating to produce browning products, one of which, acrylamide, is reported to cause numerous adverse effects in the cells, animals, and possibly also humans [55]. The analysis of amino acids is of great importance due to nutritional values and labeling requirements, control of process operating conditions, and identification of food origin as used in various products [56].

### 3.3. Bioactive Compound

**Figure 3:**
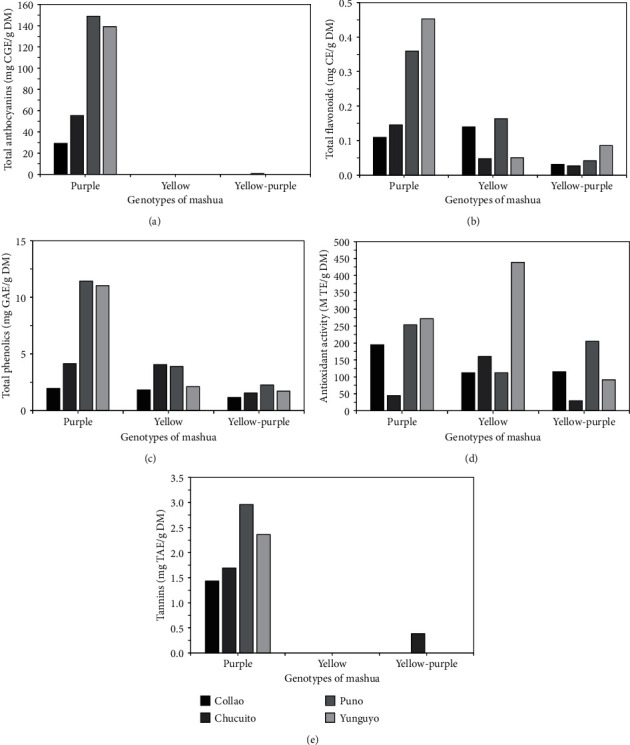
Effect of agroecological areas on the content of total anthocyanins (TA), total flavonoids (TFA), total phenolics (TP), activity antioxidant (CA), and tannins (TA) from the three mashua genotypes^∗^. ^∗^Three mashua genotypes are yellow, purple, and yellow-purple. Expressed in DM (dry matter), GAE (gallic acid equivalents), TE (Trolox equivalents), CGE (cyaniding 3-glociside equivalents), CE (catechin equivalents), TAE and (tannic acid equivalents). The means within a row with different letters are significantly different at *p* < 0.05. ND: not detected.

The contents of bioactive compounds in the three genotypes of mashua from different agroecological areas are shown in Figure 3. A significant variation was observed for the anthocyanin content in the samples. The total anthocyanin content in purple mashua ranged from 29.20 ± 0.44 to 148.90 ± 0.14 mg/g DM, while in yellow-purple, there was very little amount of total anthocyanins (0.79 ± 0.03 mg/100 g DM). However, in yellow mashua, the presence of total anthocyanins was not detected. The anthocyanin content of the purple genotype (Figure 3(a)) was similar to those obtained by Velásquez et al. [57], where they found anthocyanin concentrations of 34.58 ± 0.127 mg/g DM. These values were higher than those obtained by [5, 25, 31, 58], where they found anthocyanin concentrations of 3.7 to 8.7 mg/100 g, 11.4 to 13.6 mg/g, 0.5 to 2.05 mg/g, and 0.01 to 3.63 mg/g DM, respectively. Mashua with purple pigmentations shows higher anthocyanin content compared to genotypes with yellow pulps [5]. It seemed that anthocyanin was the main compound providing color in the darkest colored mashua landrace [31].

The total flavonoid content of three genotypes of mashua tubers from different agroecological areas is presented in Figure 3(b). This content was from 0.027 ± 0.001 to 0.453 ± 0.001 mg/g DM. A significant variation was observed for the total content of flavonoids in the samples; this was affected by the genotype and the agroecological area. The purple mashua presented the highest amount of flavonoids (0.109 ± 0.002 to 0.453 ± 0.002), followed by yellow (0.047 ± 0.00 to 0.164 ± 0.001 mg/g DM), and very little amount of total flavonoids was found in yellow-purple (0.027 ± 0.0004 to 0.086 ± 0.001 mg/g DM). The total content of flavonoids in mashua tubers was lower than those obtained by [16, 57, 59], where they found total flavonoid concentrations of 1.39 ± 0 mg/g DM, 1.8 ± 0.3 to 7.8 ± 0.4 mg CE/g extract DM, and 0.2 to 5.3 mg/g catechin equivalents DM, respectively. Flavonoid content in mashua samples depends upon the geographical origin and ecosystem [60]. The degradation of flavonoids depends on their structure, temperature, storage time, pH, oxygen, and other phytochemicals [57].

The total contents of phenolic of three genotypes of mashua tuber from different agroecological areas were from 1.16 ± 0.03 to 11.43 ± 0.05 mg/g (Figure 3(c)). A significant variation of agroecological areas on total phenolics was observed in the three genotypes. The purple mashua presented the highest amount of total phenolic (3.85 ± 0.02 to 11.43 ± 0.05 mg/g DM), followed by yellow (1.82 ± 0.03 to 4.05 ± 0.04 mg/g DM), and very little amount of total phenolic was found in yellow-purple (1.16 ± 0.03 to 2.25 ± 0.02 mg/g DM). In the agroecological zone of Chucuito, the highest amount of total phenols was found in the purple mashua. The total content of phenols in the mashua tuber was similar to those obtained by Chirinos et al. [25] who reported from 0.20 to 22.0 mg CE/g DM. The results depend on the mashua genotype, solvent type, pH, methanol/water ratio, and different acidified solvents [16].

The contents of antioxidant activity of three genotypes of mashua tuber from different agroecological areas were from 29.59 ± 0.23 to 438.56 ± 2.06 *μ*M/g DM in three genotypes from different agroecological areas (Figure 3(d)). A significant variation of agroecological areas on the content of antioxidant activity was observed in the three genotypes. The yellow mashua presented the highest amount of antioxidant activity (112.00 ± 29.51 to 438.56 ± 2.06 *μ*M/g DM), followed by purple (44.58 ± 0.12 to 272.35 ± 16.78 *μ*M/g DM), and very little amount of antioxidant activity was found in yellow-purple (29.59 ± 0.23 to 205.45 ± 1.10 *μ*M/g DM).

The purple mashua was found with the highest amount in the agroecological area of Chucuito. The total content of antioxidant activity in the mashua tuber was similar to those obtained by Chirinos et al. [25] who reported from 4 to 402 *μ*M TE/g DM and from 80 to 378 *μ*M TE/g reported by [16]. The content of antioxidant activity depends on the genotype, solvent type, pH, methanol/water ratio, and different acidified solvents. The purple mashua presented the highest ranking for antioxidant capacity [16].

The content of tannin acid was from 0.39 ± 0.02 to 2.96 ± 0.07 mg/g DM in three genotypes of mashua tuber from different agroecological areas (Figure 3(e)). The purple presented the highest amount of tannin acid (1.44 ± 0.04 to 2.96 ± 0.07 mg/g DM), followed by yellow with purple (0.39 ± 0.02 *μ*M/g DM); however, in yellow, the presence of tannin acid was not detected. Tannins are defined as phenolic compounds of high molecular matter ranging from 500 Da to more than 3000 Da which they found in plant leaves, bark, fruit, wood, and roots located basically in the tissues in the vacuoles [61]. The anticarcinogenic and antimutagenic potentials of tannins may be related to their antioxidative property, which is important in protecting cellular oxidative damage, including lipid peroxidation. The growth of many fungi, yeasts, bacteria, and viruses was inhibited by tannins [28].

## 4. Conclusions

This study demonstrated for the first time that the content of vitamins, amino acids, and bioactive compounds in mashua is influenced by ecological areas and its genotype. The yellow, purple, and yellow-purple genotypes have significant amounts of protein, fiber, phosphorus, potassium, zinc, and vitamin C. The amounts of total free amino acids in the mashua genotypes were found higher concentration aspartic acid, glutamic acid, serine, glycine, threonine, tyrosine, valine, and isoleucine. Higher amounts of total phenolic, tannins, total flavonoids, and total anthocyanins were found in purple from the agroecological areas of Yunguyo and Puno. The yellow genotypes contained a good amount of *β*-carotene and antioxidant activity. In general, these results suggested that mashua tubers grown in extreme conditions in the Peruvian Altiplano region could be interesting as a nutritious food and curative and natural source of functional ingredients for Andean communities and the world.

## Figures and Tables

**Table 1:** Physicochemical characterization of three genotypes of mashua from different agroecological areas.

Component^∗^	Genotypes	Agroecological areas^∗∗^			
		Collao	Chucuito	Puno	Yunguyo
Moisture (g/100 g FM)	Purple	89.72 ± 0.62^c^	74.51 ± 0.07^a^	84.83 ± 0.47^b^	86.72 ± 0.75^b^
	Yellow	82.86 ± 0.20^a^	86.33 ± 0.28^b^	86.90 ± 0.71^b^	87.81 ± 0.21^b^
	Yellow-purple	76.11 ± 0.42^a^	80.85 ± 0.49^b^	81.00 ± 0.35^b^	83.48 ± 0.21^c^
Protein (g/100 g DM)	Purple	11.72 ± 0.05^d^	7.41 ± 0.01^a^	7.86 ± 0.01^b^	8.16 ± 0.06^c^
	Yellow	7.83 ± 0.03^b^	7.58 ± 0.08^b^	6.96 ± 0.09^a^	9.98 ± 0.02^c^
	Yellow-purple	7.54 ± 0.03^b^	7.15 ± 0.04^a^	7.31 ± 0.09^a^	7.95 ± 0.04^c^
Fat (g/100 g DM)	Purple	4.53 ± 0.06^ab^	4.40 ± 0.13^a^	4.57 ± 0.02^ab^	4.70 ± 0.01^b^
	Yellow	4.60 ± 0.12^a^	5.63 ± 0.02^b^	4.70 ± 0.05^a^	4.79 ± 0.01^a^
	Yellow-purple	4.567 ± 0.06^a^	4.62 ± 0.06^a^	4.57 ± 0.07^a^	4.71 ± 0.04^a^
Ash (g/100 g DM)	Purple	6.66 ± 0.08^c^	5.32 ± 0.05^a^	5.84 ± 0.13^b^	6.40 ± 0.03^c^
	Yellow	7.18 ± 0.08^b^	5.22 ± 0.35^a^	5.60 ± 0.07^a^	5.23 ± 0.05^a^
	Yellow-purple	5.23 ± 0.09^c^	4.81 ± 0.09^b^	5.29 ± 0.01^c^	4.12 ± 0.02^a^
Fiber (g/100 g DM)	Purple	6.36 ± 0.03^b^	5.89 ± 0.02^a^	5.78 ± 0.14^a^	5.79 ± 0.10^a^
	Yellow	5.99 ± 0.12^a^	5.93 ± 0.03^a^	5.93 ± 0.04^a^	6.2 ± 0.06^a^
	Yellow-purple	5.23 ± 0.03^a^	5.91 ± 0.06^b^	5.85 ± 0.04^b^	5.79 ± 0.13^b^
Carbohydrate (g/100 g DM)	Purple	70.73 ± 0.16^a^	76.99 ± 0.21^d^	75.96 ± 0.30^c^	74.96 ± 0.02^b^
	Yellow	74.40 ± 0.11^a^	75.64 ± 0.31^b^	76.82 ± 0.25^c^	73.79 ± 0.07^a^
	Yellow-purple	77.33 ± 0.03^a^	77.50 ± 0.17^a^	76.98 ± 0.20^a^	77.43 ± 0.06^a^
Calcium (mg/100 g DM)	Purple	53.32 ± 2.85^c^	40.38 ± 1.18^ab^	44.95 ± 1.52^b^	35.66 ± 0.26^a^
	Yellow	51.34 ± 1.06^c^	38.21 ± 1.05^a^	45.39 ± 1.58^b^	35.61 ± 0.53^a^
	Yellow-purple	37.81 ± 0.55^b^	35.98 ± 0.61^ab^	46.89 ± 0.55^c^	34.78 ± 0.86^a^
Phosphorus (mg/100 g DM)	Purple	191.55 ± 8.36^c^	146.95 ± 5.15^a^	166.69 ± 4.89^ab^	179.98 ± 2.61^bc^
	Yellow	179.31 ± 3.40^b^	142.47 ± 3.82^a^	155.92 ± 8.37^a^	143.24 ± 2.57^a^
	Yellow-purple	139.90 ± 2.68^b^	132.18 ± 3.08^b^	138.97 ± 3.29^b^	114.56 ± 0.44^a^
Iron (mg/100 g DM)	Purple	7.74 ± 0.39^a^	7.60 ± 0.01^a^	76.85 ± 0.35^a^	7.57 ± 0.15^a^
	Yellow	7.61 ± 0.12^a^	7.81 ± 0.45^a^	7.51 ± 0.04^a^	7.60 ± 0.14^a^
	Yellow-purple	7.36 ± 0.06^a^	7.66 ± 0.38^a^	7.22 ± 0.07^a^	7.02 ± 0.17^a^
Potassium (mg/100 g DM)	Purple	1767.26 ± 19.13^a^	1875.61 ± 15.38^a^	1829.78 ± 32.29^a^	1729.18 ± 32.77^a^
	Yellow	1789.28 ± 7.47^a^	2021.15 ± 5.45^b^	1723.42 ± 54.33^a^	1773.84 ± 19.57^a^
	Yellow-purple	1743.54 ± 9.13^a^	1789.77 ± 11.64^a^	1751.92 ± 23.92^a^	1781.32 ± 25.91^a^
Zinc (ppm)	Purple	9.940 ± 0.08^d^	7.41 ± 0.16^b^	8.42 ± 0.05^c^	4.11 ± 0.10^a^
	Yellow	11.63 ± 0.03^d^	7.25 ± 0.02^c^	4.15 ± 0.03^a^	6.11 ± 0.10^b^
	Yellow-purple	11.99 ± 0.06^c^	6.11 ± 0.02^b^	4.53 ± 0.04^a^	6.18 ± 0.05^b^
*β*-Carotene (*μ*g/g DM)	Purple	0.00 ± 0.00^a^	5.65 ± 0.8^b^	0.00 ± 0.00^a^	0.00 ± 0.00^a^
	Yellow	89.58 ± 1.180^b^	715.95 ± 84.03^d^	613.99 ± 1.61^c^	18.10 ± 0.13^a^
	Yellow-purple	336.33 ± 3.21^d^	13.70 ± 0.44^c^	6.91 ± 0.03^a^	15.09 ± 0.19^b^
Vitamin C (mg/g DM)	Purple	2.63 ± 0.01^c^	4.46 ± 0.01^d^	1.31 ± 0.0004^b^	1.21 ± 0.002^a^
	Yellow	1.54 ± 0.001^d^	1.17 ± 0.01^c^	0.95 ± 0.001^b^	0.53 ± 0.002^a^
	Yellow-purple	0.90 ± 0.003^a^	3.36 ± 0.002^d^	1.32 ± 0.003^b^	1.76 ± 0.002^c^

^∗^Expressed in FM (fresh matter) and DM (dry matter). ^∗∗^The results are presented as the SD of the means. Means with different superscripts (alphabets) in the same column are significantly different, *p* < 0.05.

**Table 2:** Effect of agroecological areas on the content of amino acids (mg/g DM) from the three mashua genotypes^∗^.

Amino acid	Mashua genotypes	Agroecological areas^∗∗^			
		Collao	Chucuito	Puno	Yunguyo
Aspartic acid	Purple	0.11 ± 0.08^a^	0.329 ± 0.014^bc^	0.275 ± 0.01^b^	0.438 ± 0.04^c^
	Yellow	0.344 ± 0.02^a^	0.340 ± 0.04^a^	0.334 ± 0.02^a^	0.792 ± 0.05^b^
	Yellow-purple	0.264 ± 0.02^b^	0.185 ± 0.01^a^	0.348 ± 0.03^c^	0.364 ± 0.001^c^
Glutamic acid	Purple	0.30 ± 0.01^b^	0.346 ± 0.009^c^	0.226 ± 0.01^a^	0.302 ± 0.01^b^
	Yellow	0.364 ± 0.02^ab^	0.329 ± 0.02^a^	0.417 ± 0.03^b^	0.549 ± 0.02^c^
	Yellow-purple	0.305 ± 0.02^bc^	0.264 ± 0.02^b^	0.219 ± 0.01^a^	0.306 ± 0.05^c^
Serine	Purple	0.47 ± 0.01^b^	0.382 ± 0.006^a^	0.361 ± 0.01^a^	0.479 ± 0.02^b^
	Yellow	0.456 ± 0.01^a^	0.475 ± 0.02^ab^	0.515 ± 0.02^b^	0.745 ± 0.02^c^
	Yellow-purple	0.395 ± 0.01^b^	0.356 ± 0.02^a^	0.371 ± 0.02^ab^	0.400 ± 0.01^b^
Histidine	Purple	0.08 ± 0.001^c^	0.094 ± 0.01^c^	0.052 ± 0.01^a^	0.071 ± 0.002^b^
	Yellow	0.120 ± 0.08^c^	0.075 ± 0.04^a^	0.074 ± 0.01^a^	0.096 ± 0.01^b^
	Yellow-purple	0.104 ± 0.01^b^	0.071 ± 0.01^b^	0.059 ± 0.004^ab^	0.049 ± 0.004^a^
Glycine	Purple	0.33 ± 0.01^c^	0.303 ± 0.002^b^	0.236 ± 0.01^a^	0.308 ± 0.01^b^
	Yellow	0.392 ± 0.005^a^	0.398 ± 0.02^a^	0.428 ± 0.02^ab^	0.445 ± 0.02^b^
	Yellow-purple	0.367 ± 0.01^c^	0.274 ± 0.004^a^	0.264 ± 0.003^a^	0.298 ± 0.01^b^
Threonine	Purple	0.51 ± 0.01^d^	0.336 ± 0.03^a^	0.382 ± 0.01^b^	0.468 ± 0.02^c^
	Yellow	0.440 ± 0.01^a^	0.485 ± 0.01^b^	0.504 ± 0.02^b^	0.694 ± 0.01^c^
	Yellow-purple	0.369 ± 0.01^a^	0.374 ± 0.01^a^	0.376 ± 0.01^a^	0.406 ± 0.003^b^
Arginine	Purple	0.16 ± 0.004^a^	0.334 ± 0.08^c^	0.135 ± 0.02^a^	0.210 ± 0.01^b^
	Yellow	0.592 ± 0.01^b^	0.193 ± 0.13^a^	0.264 ± 0.02^a^	0.340 ± 0.005^a^
	Yellow-purple	0.419 ± 0.02^c^	0.245 ± 0.01^b^	0.159 ± 0.004^a^	0.152 ± 0.01^a^
Alanine	Purple	0.14 ± 0.02^bc^	0.123 ± 0.02^ab^	0.116 ± 0.004^a^	0.147 ± 0.004^c^
	Yellow	0.406 ± 0.01^c^	0.158 ± 0.05^a^	0.173 ± 0.01^a^	0.249 ± 0.02^b^
	Yellow-purple	0.257 ± 0.01^b^	0.117 ± 0.01^a^	0.123 ± 0.01^a^	0.129 ± 0.002^a^
Tyrosine	Purple	0.54 ± 0.01^a^	0.263 ± 0.07^c^	0.382 ± 0.01^ab^	0.452 ± 0.003^b^
	Yellow	0.403 ± 0.01^c^	0.464 ± 0.01^a^	0.472 ± 0.01^a^	0.576 ± 0.004^b^
	Yellow-purple	0.303 ± 0.01^a^	0.325 ± 0.004^b^	0.352 ± 0.002^c^	0.391 ± 0.01^d^
Cystine	Purple	ND	ND	ND	ND
	Yellow	ND	ND	ND	ND
	Yellow-purple	ND	ND	ND	ND
Valine	Purple	0.43 ± 0.01^a^	0.328 ± 0.05^a^	0.321 ± 0.02^a^	0.393 ± 0.01^a^
	Yellow	0.105 ± 0.01^a^	0.358 ± 0.03^a^	0.317 ± 0.001^a^	0.422 ± 0.01^a^
	Yellow-purple	0.241 ± 0.06^a^	0.289 ± 0.01^a^	0.283 ± 0.01^a^	0.219 ± 0.002^a^
Methionine	Purple	ND	ND	0.106 ± 0.18^a^	0.115 ± 0.02^a^
	Yellow	ND	ND	0.128 ± 0.02^a^	0.139 ± 0.02^a^
	Yellow-purple	ND	ND	ND	ND
Phenylalanine	Purple	0.10 ± 0.002^a^	0.102 ± 0.04^a^	0.092 ± 0.002^a^	0.119 ± 0.01^b^
	Yellow	0.105 ± 0.01^a^	0.169 ± 0.001^c^	0.127 ± 0.01^b^	0.205 ± 0.005^d^
	Yellow-purple	0.099 ± 0.01^b^	0.089 ± 0.003^ab^	0.091 ± 0.004^ab^	0.080 ± 0.003^a^
Isoleucine	Purple	0.84 ± 0.03^a^	0.604 ± 0.012^b^	0.649 ± 0.01^a^	0.820 ± 0.02^b^
	Yellow	0.732 ± 0.08^a^	0.821 ± 0.01^a^	0.772 ± 0.03^a^	1.086 ± 0.02^b^
	Yellow-purple	0.552 ± 0.02^a^	0.584 ± 0.02^ab^	0.683 ± 0.02^c^	0.612 ± 0.002^b^
Leucine	Purple	0.14 ± 0.004^a^	0.173 ± 0.05^b^	0.124 ± 0.01^a^	0.178 ± 0.01^b^
	Yellow	0.190 ± 0.003^a^	0.187 ± 0.004^a^	0.197 ± 0.01^a^	0.297 ± 0.005^b^
	Yellow-purple	0.179 ± 0.01^b^	0.140 ± 0.01^a^	0.137 ± 0.009^a^	0.142 ± 0.01^a^
Lysine	Purple	0.18 ± 0.004^c^	0.143 ± 0.05^a^	0.135 ± 0.01^a^	0.159 ± 0.01^b^
	Yellow	0.208 ± 0.01^a^	0.210 ± 0.02^a^	0.213 ± 0.01^a^	0.191 ± 0.01^a^
	Yellow-purple	0.179 ± 0.01^b^	0.163 ± 0.01^ab^	0.153 ± 0.004^a^	0.156 ± 0.01^a^
Proline	Purple	ND	ND	ND	ND
	Yellow	ND	ND	ND	ND
	Yellow-purple	ND	ND	ND	ND
Total amino acid	Purple	4.45 ± 0.194	3.859 ± 0.082	3.592 ± 0.320	4.659 ± 0.355
	Yellow	2.763 ± 0.42	4.664 ± 0.29	4.935 ± 0.43	6.825 ± 0.45
	Yellow-purple	4.032 ± 0.232	3.478 ± 0.132	3.617 ± 0.147	3.704 ± 0.091

^∗^Three mashua genotypes are yellow, purple, and yellow-purple. Expressed in DM (dry matter). ^∗∗^The means within a row with different letters are significantly different at *p* < 0.05. ND: not detected.

## Data Availability

Table and figure data used to support the conclusions of this study are included in the article.
